# Determining the efficacy of national strategies aimed at addressing the challenges facing health personnel working in rural areas in KwaZulu-Natal, South Africa

**DOI:** 10.4102/phcfm.v9i1.1355

**Published:** 2017-07-31

**Authors:** Grace Mburu, Gavin George

**Affiliations:** 1 School of Management, Information Technology & Governance, University of KwaZulu-Natal, South Africa; 2 Health Economics and HIV and AIDS Research Division (HEARD), University of KwaZulu-Natal, South Africa

## Abstract

**Background:**

Shortages of Human Resources for Health (HRH) in rural areas are often driven by poor working and living conditions, inadequate salaries and benefits, lack of training and career development opportunities amongst others. The South African government has adopted a human resource strategy for the health sector in 2011 aimed at addressing these challenges.

**Aim:**

This study reviews the challenges faced by health personnel against government strategies aimed at attracting and retaining health personnel in these underserved areas.

**Setting:**

The study was conducted in six primary health care service sites in the Hlabisa sub-district of Umkhanyakude, located in northern KwaZulu-Natal, South Africa.

**Methods:**

The study population comprised 25 health workers including 11 professional nurses, 4 staff nurses and 10 doctors (4 medical doctors, 3 foreign medical doctors and 3 doctors undertaking community service). Qualitative data were collected from semi-structured interviews and analysed using thematic analysis.

**Results:**

Government initiatives including the rural allowance, deployment of foreign medical doctors and the presence of health personnel undertaking their community service in rural areas are positively viewed by health personnel working in rural health facilities. However, poor living and working conditions, together with inadequate personal development opportunities, remain unresolved challenges. It is these challenges that will continue to dissuade experienced health personnel from remaining in these underserved areas.

**Conclusion:**

South Africa’s HRH strategy for the Health Sector 2012/13–2015/16 had highlighted the key challenges raised by respondents and identified strategies aimed at addressing these challenges. Implementation of these strategies is key to improving both living and working conditions, and providing health personnel with opportunities for further development will require inter-ministerial collaboration if the HRH 2030 objectives are to be realised.

## Introduction

Inadequate Human Resources for Health (HRH) has been identified globally as a major hindrance to the delivery of quality health services^1^. This is a contributing factor to most developing countries’ inability to achieve the health-related millennium development goals^2,3^ and will undoubtedly factor in the achievement of the sustainable development goals. In Africa, the shortage of HRH is more prevalent in rural areas, which puts an incredible strain on the health care system, resulting in preventable deaths and an increased burden of disease^4^. This raises concerns, especially considering South Africa has the world’s largest HIV epidemic with an estimated seven million infected individuals^5^. This high disease burden has placed pressure on health facilities, resulting in the need to increase the health care workforce in order to alleviate the increasing workload, especially in the rural areas^6^.

There exists a doctor and nurse deficit within the public health sector for manifold reasons: training and recruitment has not kept abreast of population growth, staff attrition and the increasing burden of care imposed on the health system by the large and growing HIV and AIDS epidemic in the country. Furthermore, disparities in HRH between the public and private sectors and rural and urban areas have dramatically increased, serving to transform ‘health’ into a commodity rather than a human right^7^.

In 2010, an estimated 30% of South Africa’s medical practitioners worked in the public sector where they served an estimated 84% of the population^8^. This disparity is acutely illustrated by the distribution of medical specialists in the private sector which was 86.5 per 100 000 beneficiaries, while only 5.6 specialists per 100 000 within the public sector–dependent population in 2013^9^. The South African Department of Health (DoH) estimated a shortfall of approximately 80 000 medical practitioners in 2011, which translated into a 56% vacancy rate for doctors and 46% vacancy rate for nurses in the public health sector^6^.

Furthermore, the distribution of HRH within South Africa between urban and rural areas is notably uneven, largely because of problems of attracting and retaining health workers to rural areas. It is estimated that 46% of the population live in rural areas but are served by only 12% of doctors and 19% of nurses^10,11^. Rural provinces, for instance, the Northern Cape, Eastern Cape, Mpumalanga and Limpopo, had the lowest number of medical specialists per 100 000 in 2014 with 3.1, 2.8, 2.0 and 1.8, respectively^9^. Conversely, provinces that host the country’s major cities like Gauteng and Western Cape have a much higher number of medical specialists per population ratio with 20.5 and 31.3, respectively. The same trends are seen amongst registered nurses in rural provinces; for example, Mpumalanga, Northern Cape and Limpopo had density ratios of 165, 192 and 195 per 100 000, respectively, in 2014 versus their urban counterparts that experienced density ratios of 270 per 100 000 both in Gauteng and the Western Cape^12^.

The shortage of HRH in the rural areas is largely because of the inability to attract sufficient health personnel to these communities and further because of migration from the rural to urban areas^13^. Migration is fuelled by the challenges faced by HRH and hence their decision to leave the rural areas. These challenges have previously included inadequate salaries and wages, poor and unsafe working conditions, lack of career development opportunities, lack of management support and supervision, work overload, emotional burnout, inadequate resources and poor infrastructure^6,7,13,14^. In 2011, the South African government launched the HRH strategy for the Health Sector 2012/13–2015/16, aimed at addressing the shortage of health personnel working in the rural health care system. Strategies aimed to correct the imbalance of HRH between rural and urban areas sought to increase the appeal of working in the rural public health sector through the improvement of working conditions and remuneration packages of HRH.

Managing a scarce resource, such as health workers, requires a strategic approach in sourcing, attracting, selecting, training, developing and retaining health workers^15^. Neal and Gebauer^16^ further suggest that strategies to attract, retain and engage human resources should be focused on a competitive base pay, career advancement opportunities, learning and development opportunities, and good supervision and management. This study examines the challenges facing health workers in a rural area in KwaZulu-Natal, South Africa, and the perceived effect national strategies have had in attracting and retaining health personnel to underserved areas.

## Research method and design

### Research design

Data were collected by one interviewer through in-depth interviews using a semi-structured interview schedule with health personnel working in rural health facilities. The interviewer was female, with a Master’s degree and resided in the area at the time of the study.

### Study setting

The study was conducted in the sub-district of Hlabisa in KwaZulu-Natal, South Africa.

### Study population and sampling strategy

Six rural health facilities were selected based on the number of patients that were seen on a monthly basis, that is, low, medium and high, with two selected from each category. Additionally, geographic location was considered to ensure that facilities in deeper rural areas as well as those close to the main road were included. Purposive sampling was employed to ensure the health professionals selected represented a broad spectrum of health cadres. A total of 25 health personnel were interviewed, comprising 11 professional nurses, 4 staff nurses and 10 doctors including 3 foreign medical doctors and 3 doctors undertaking their community service.

### Data collection

An interview schedule was formulated in accordance with the study objectives. Firstly, data were ordered into systems and human resource challenges. Secondly, respondents were probed on the effect of government intervention strategies aimed at attracting and retaining health personnel to rural areas.

Written permission to undertake the study within the selected clinics was received from the Hlabisa sub-district HIV and AIDS health programme manager and the Hlabisa sub-district hospital management. Following approval, the selected clinics were visited with available HRH (nurses and doctors) informed of the objectives of the study. Upon receipt of consent to participate, appointments were made for face-to-face interviews. The researcher (and primary author) conducted all the interviews with participants at the clinics behind closed doors to ensure privacy. All interviews were audio recorded and transcribed by the same researcher.

### Data analysis

Following accepted good practice, transcripts were read twice before coding^17^. Data were coded into ‘expressions’ and ‘themes’. Descriptive codes were used to identify basic expressions that were found in individual transcripts. These were compared with other expressions emerging from the interviews and, after being categorised into sub-themes, were assigned further analytical coding labels. Qualitative techniques for identifying themes from basic expressions in the text were used as recommended by Ryan and Bernard^18^. The process of analysing the data moved from descriptive to analytical coding as guided by Gibbs^17^.

Comparative analysis was undertaken to ensure that the codes developed from the data were consistent and accurately applied throughout the analysis to ensure that similar data were uniformly coded^17^.

### Ethical consideration

Ethical approval for this study was obtained from the University of KwaZulu-Natal’s Humanities and Social Sciences Research Ethics Committee (HSS/1236/011M). Participants were informed that their participation in the study was voluntary and assured of their right to withdraw at any time. Recordings of the interviews were kept on a single, password protected computer. Transcripts were kept in a locked filing cabinet only accessed by the researcher. The manuscript reveals no identifying information of the participants or the clinics ensuring confidentiality of the participant’s views.

## Results

Health personnel emphasised the need for government to intervene and improve working conditions in rural health facilities in order to attract and retain staff. The challenges reported by respondents are limited to those that affect the optimal delivery of health services.

Challenges have been divided into system and human resource challenges.

The final section covers national policies identified by respondents as impacting on their working conditions. Respondents provided comment on the perceived effectiveness of these policies and highlight unresolved issues.

### Health system challenges

#### Infrastructure

Exacerbating the high disease burden is the lack of basic infrastructure such as available water and electricity:

‘We have a problem with water and electricity so some doctors and nurses don’t want to work here. Like today you can see we are not busy, because we don’t have electricity to operate the equipment.’ (Participant 7, Female, Staff Nurse)

‘If I can tell you we don’t have water since Monday, we don’t have electricity since the morning and we don’t have generators. In the front we need to do vital signs, but all the equipment’s work with electricity so we can’t do much.’ (Participant 11, Female, Professional Nurse)

‘The roads to most of the clinics are generally ok. However it’s a nightmare driving to the deep rural clinics.’ (Participant 23, Male, Foreign Medical Doctor)

#### Equipment and supplies

Participants suggested that there was simply inadequate equipment and medication available in certain high volume clinics. Specifically, some of the TB wards did not have masks and infectious disease control medication for health professionals. The procurement of medication and other medical supplies was often delayed:

‘We don’t have equipment, we are overcrowded and we end up using one BP machine for like 1000 people, so if there’s no electricity, even the scales won’t work. Our equipment is bad. There are no thermometers if a baby comes with fever, you must use your hand.’ (Participant 7, Female, Staff Nurse)

‘Supplies would be the main problem, they are there but not enough in clinics and we are very frustrated.’ (Participant 4, Female, Professional Nurse)

‘With the supplies, when it’s finished there are delays, but generally the government is trying, the only problem is that the patients have to come back and fetch the medicine and they are coming from very far.’ (Participant 3, Female, Professional Nurse)

‘Sometimes when the patients come we don’t have medicine for them because of the delays so we ask them to come back after a few days to collect. You find when the patient comes back they are really sick, with multiple symptoms hence you have to diagnose again.’ (Participant 17, Male, Medical Doctor)

#### Referral systems

Doctors and nurses were particularly concerned with prevailing referral systems. Because of the administrative burden, doctors were left frustrated at the process of referring patients and ensuring they received the appropriate treatment:

‘The referral path right now doesn’t work; it’s very difficult to refer a sick patient to a higher level (facility). What happens is that Albert Luthuli and King Edward (Provincial Hospitals) are so far away from us, it takes me one day of my time to try get a patient through to one of the facilities if I’m lucky, sometimes it takes even more.’ (Participant 17, Male, Medical Doctor)

‘The referral system is completely broken; you can spend the whole time on the phone trying to sort out something.’ (Male, Foreign Medical Doctor)

‘With us nurses we can refer a person to the hospital only to find they are left for days and days because of the overload of patients, only to come back and say I went there but I didn’t get any help so I came back.’ (Participant 11, Female, Professional Nurse)

#### Culture

Tension between the administration of allopathic medicine and the presence of traditional medicine prevailed within the rural setting. Cultural beliefs and practices often hindered efforts to provide prescribed health care:

‘Due to ignorance and also culture – they believe they are bewitched, it will take I don’t know how many ages for us to get away from the culture and get enlightened. Some people are even highly educated but when you sit down with them you find that culture is deeply rooted in our blood, we have been mentored with beliefs.’ (Participant 1, Female, Professional Nurse)

‘Culture is a problem for most of them (patients), because they still deeply believe in our cultures, especially with ART it’s even worse, that’s why we had this resistance and patients defaulting from taking medication, because we still believe so much in culture.’ (Participant 9, Female, Professional Nurse)

‘One main challenge for me is the conflict between traditional and modern medicine. But you understand it’s an effect of culture.’ (Participant 23, Male, Foreign Medical Doctor)

### Human resource management challenges

#### High workload

Because of inadequate HRH, health personnel attend to a high volume of patients on a daily basis, and because most of these patients are coming from far, they have to be attended to on the same day to avoid a backlog. This often leads to emotional burnout, stress and frustrations, as expressed below:

‘There is definitely a challenge of dealing with the burden of disease, HIV and TB, there’s just so much that it’s stretching the health system, the health system can’t really cope. We are working under pressure, and working really hard but there’s no light at the end of tunnel.’ (Participant 18, Male, Foreign medical Doctor)

‘Working under pressure and working hard but there’s no light at the end of tunnel.’ (Participant 23, Male, Foreign Medical Doctor)

‘The workload is too much; imagine you are supposed to do a job of three people.’ (Participant 5, Female, Professional Nurse)

‘The work overload is unbearable, it’s too much, and we need more nurses and doctors to help.’ (Participant 20, Female, Professional Nurse)

‘We experience emotional burnout, you say something to someone, not intentionally but because you are tired, only to regret later, I wasn’t aware I hurt someone.’ (Participant 14, Female, Professional Nurse)

‘I think as staff we need to have a plan for the staff like to refresh them, maybe even just once a year. I think that helps you a lot, take nurses out to help them take out the steam, it keeps you going.’ (Participant 19, Female, Professional Nurse)

‘We get really drained emotionally. No time for lunch especially here in TB [*Tuberculosis Ward*] you get tired.’ (Participant 2, Female, Staff Nurse)

The support of management is viewed as critical in ensuring that health personnel are working to optimal levels.

#### Management support and supervision

There were mixed feelings with regard to the management and supervision offered at health facilities. Health personnel working in deep rural areas felt that they had been neglected by management, which only responded in a crisis situation:

‘Most of the time nobody appreciates us from management, we work in the clinics; meaning we have to handle so much in the clinics. So I believe recognition is important.’ (Participant 4, Female, Professional Nurse)

‘You see when you are working at the clinics you just feel like you are deserted. Management will only come when there is a crisis.’ (Participant 19, Female, Professional Nurse)

Health personnel called for a forum through which to voice their dissatisfactions and consequently expected timely and open feedback from management:

‘The channels of communication are not all that reliable, sometimes as a nurse you want to suggest something or say something, but then you find that there’s a barrier between the unit manager and nurse manager.’ (Participant 1, Female, Professional Nurse)

‘The problem is that even when the people from the Department of Health come to the community to address them, they do not prioritise as to what is lacking in the clinics, there is no proper feedback on the challenges on the ground.’ (Participant 8, Female, Professional Nurse)

#### Professional development opportunities

Professional development was important and even more critical in rural areas where task-shifting strategies are adopted because of the scarcity of health professionals, especially doctors. Respondents identified a need to be empowered with additional skills to enable them to deliver quality health care to their patients:

‘The government should consider the nurses who work in the clinics and consider empowering them since at the clinics we haven’t got doctors and have to make our own decisions, and if anything goes wrong it’s your baby, you have to deal with the situation the way it is. We need to be empowered with skills and workshops that give us more training and education.’ (Participant 11, Female, Professional Nurse)

‘We need professional development because you are isolated out here, but especially if referral hospitals would encourage that. Some kind of interaction will make you feel like they are supporting you.’ (Participant 12, Male, Community Service Doctor)

‘You find that no one is offering you the skills and development of what the rural doctors don’t know so you also end up becoming another rural doctor.’ (Participant 23, Male, Community Service Doctor)

#### Availability of doctors and specialists

Participants were of the opinion that health facilities benefited from the presence of supplementary medical doctors, even if they were only available periodically. The lack of available specialists was a concern but respondents felt that the introduction of District Clinical Specialist Teams could potentially alleviate this problem:

‘Doctors used to come once a week but they used to come from another hospital. We have doctors who come on Tuesdays, however, they used to be helped by NGO doctors who are specialised with HIV, and otherwise many people would have died. Especially with the children, even today there is a doctor who comes on a Friday, if you look over there she even comes with toys, children are playing, even the children who don’t have opportunity to play with the toys are very happy.’ (Participant 5, Female, Professional Nurse)

‘The government is trying to address this problem by introducing the District Support Specialists [*District Clinical Specialist Teams*]^1^, the strategy implementation is underway, and this would solve the specialist problem to a great extent.’ (Participant 17, Male, Medical Doctor)

### National policies aimed at attracting and retaining health professionals in underserved areas

The strategies identified by respondents to have made an impact on health personnel working in the rural areas include the rural allowance, community service for health professionals and the recruitment of foreign medical doctors to work in underserved areas. Health personnel discussed the efficacy of these policy initiates and their effect on addressing the challenges facing health professionals working in rural areas.

#### Rural allowance

Most of the nurses welcomed the initiative by government to introduce rural allowances as this was one way to make them feel appreciated. Nurses felt that the rural allowance constituted a real incentive to work in rural over urban centres:

‘We are so happy about it. It has improved our life, and the way they have done it, we are so happy, it improves our lives.’ (Participant 3, Female, Professional Nurse)

‘People are keen to move from NGOs back to DoH, because the salaries are better in DoH, so it’s clear to me that strategies are definitely working, they are encouraging people from NGOs back to DoH which is a good thing. It’s working to an extent.’ (Participant 18, Male, Foreign Medical Doctor)

However, respondents felt that there was an inequitable distribution of rural allowances and that the criteria used in the definition of what constituted ‘rural’ were problematic. Some respondents provided examples of what they consider to be the unfair distribution stating that, for instance, some personnel in hospitals in rural areas get an 18% allowance, while others in Port Shepstone, which is a fairly modern town with shopping malls and cinemas, get the maximum of 22%. This suggests that the current classification criteria are inadequate in distinguishing from deep rural, rural and peri-urban settings:

‘So if you want to incentivise it, award doctors working in deep rural significantly more than others in less rural areas, not asking for lots of money but it needs to be reasonable.’ (Participant 21, Male, Medical Doctor)

‘Rural allowance lacked proper definition which has resulted in discrepancies in its administration. Some doctors in peri-urban areas are getting about 22% while some of us working in rural and deep rural would only get 18%.’ (Participant 17, Male, Medical Doctor)

Furthermore, staff nurses working in rural areas are not entitled to a rural allowance despite facing the same challenges as their more qualified colleagues. Staff nurses therefore felt that the rural allowance bred animosity amongst health personnel as only certain health cadres were afforded this benefit. They called for the benefit to be standardised across the board:

‘I’m not happy about rural allowances. We are all working in a rural area, but when it comes to the allowance they give priority to registered [*professional*] nurses only.’ (Participant 2, Female, Staff Nurse)

#### Recruitment of foreign medical doctors

Foreign medical doctors were recruited to underserved areas as a strategy to offset the skill shortage in rural areas. The respondents were generally positive in their views of foreign medical doctors. Respondents felt that foreign medical doctors played a positive role within the rural health system as expressed in the statements below:

‘Truly speaking, the foreign medical doctors are more dedicated compared to our doctors. They really work since they don’t have families here so they dedicate their whole time. They don’t mind working even after hours, unlike our doctors.’ (Participant 9, Female, Professional Nurse)

‘Our doctors here they don’t want to come back and work here, they want big cities where life is easier, but foreign medical doctors are willing.’ (Participant 10, Female, Professional Nurse)

‘They are good and they care, I won’t lie, they like their patients. I don’t have to compare with our doctors but I must say. Yes, they like their patients and they take a good care of their patients.’ (Participant 1, Female, Professional Nurse)

The participants were in agreement that foreign medical doctors alleviate the shortage of HRH in rural areas. Foreign medical doctors did, however, admit to challenges working in rural South Africa, especially because of the high burden of disease. The foreign medical doctors declared two main challenges they encountered: language and culture. Foreign medical health personnel would have to resort to using a translator and found it difficult to engage with patients:

‘The main challenge for me is the language, not being able to speak the language of the patients, it’s a big challenge to providing health care as a doctor, not understanding and know how to deal with issues, since you can’t properly engage with patients.’ (Participant 18, Male, Foreign Medical Doctor)

‘There’s no deep understanding of culture, as a doctor you need to understand issues that patients might have, you find yourself in the dark about some things, hence it’s a bit of a challenge since you don’t know how to deal with certain situations.’ (Participant 18, Male, Foreign Medical Doctor)

#### Community service and conditional scholarships

Community service is a government initiative requiring health professionals to serve a mandatory one year, post completion of their studies, in a district, tertiary or regional health facility. There is a lot of competition as most health professionals prefer to be posted in urban centres, where they can further their skills by obtaining specialised training, leaving the rural areas to the ‘unlucky’ few, who have no alternative. However, some health professionals are obligated by conditional scholarships which dictate that they go back and serve their community service in rural areas:

‘I’m here because of the scholarship, as one of the requirements is to come back to rural hospitals.’ (Participant 13, Female, Community Service Doctor)

‘I applied to come here because of the scholarship, and its mandatory for you to come back, the scholarship condition is for two years.’ (Participant 12, Male, Community Service Doctor)

Participants noted that the impact of community service in the rural area was evident. The sub-district hospital had seven doctors who were undertaking their community service:

‘The sub-district hospital is staffed with community service doctors, this strategy by the government definitely works, the hospital wouldn’t survive without that system, the hospital has something between 12 to 14 doctors, the majority of them are community service staff, but of course the challenge is to retain them after the fixed period of time.’ (Participant 10, Male, Medical Doctor)

## Discussion

In 2011, the DoH launched HRH 2030, a five-year HRH strategy for the health sector^6^. The strategy recognised the unique HRH challenges facing the rural health sector and identified a number of initiatives aimed at addressing them. These data have confirmed that a number of these initiatives are positively viewed by health personnel working in rural areas. Chief amongst these initiatives is the rural allowance. The implementation of the rural allowance provided an additional 18%–22% of salary for doctors and 8%–12% for professional nurses, which has resulted in creating a significant financial incentive for these health cadres to work in rural areas. However, the rural allowance has come in for some criticism around the classification of rural health facilities, and this has created consternation amongst health personnel. The Rural Health Advocacy Project has argued the importance of determining objectively the ‘rurality and remoteness’ of facilities, which would inform the rural allowance and other incentive-based initiatives^19^. Only select health personnel cadres receive the rural allowance and this too has created unhappiness amongst health personnel. Literature^20^ has cautioned that while financial incentives are important, they do not adequately compensate for other, particularly systemic, challenges facing health personnel working in rural areas.

The deployment of foreign medical doctors to rural areas along with the constant stream of health personnel undertaking community service has noticeably augmented the permanent health staff and has been generally well received by colleagues and the community.

However, the study highlights continued challenges facing HRH in rural KwaZulu-Natal against the backdrop of these policy initiatives aimed at addressing them. Poor living conditions coupled with sub-par health infrastructure including inadequate medical supplies, lack of infectious disease control measures, poor supervision and management and inadequate referral systems have resulted in poor working conditions. Government strategies have failed to address these health system challenges described by respondents. Previous studies^7^ show that there is correlation between the quality of working and living conditions and the decision to work in rural areas. Strategies implemented abroad, aimed at addressing system issues, offer examples of how improved general infrastructure (roads, phones, water supply and radio communication), together with staff housing at rural district hospitals, brought about higher rates of retention and attracted health personnel to underserved areas^21^.

Management and supervision is also a key component in realising the potential of any workforce. This study supports other findings which show that although infrastructure and equipment were reported as challenges, the need to feel valued and supported was much greater^22,23^. A study conducted in Benin and Kenya shows that HRH referred to ‘professional satisfaction’ and ‘recognition by supervisors’ as key components in ensuring retention^24^. Neuhaser^25^ affirms that management and supervision determines 50% of work–life satisfaction, which means that good management directly contributes to the decision of health personnel to stay or leave.

Finally, health personnel expressed the need for professional networks and saw value in engaging with senior health staff. These opportunities were limited within a rural setting. Studies^22,26^ reveal how professional support networks are an important component of nurses’ decisions to work in rural areas. The WHO recommends the need for professional support as a way of overcoming isolation of health personnel working in remote areas. This can be achieved if proper structures exist to ensure communication and peer consultations occur through established networks^2^. Implementation of District Support Specialist Teams could be an effective way of enhancing professional networks by having specialists available at the sub-district level.

Linked to the engagement with more senior health cadres is the desire for professional development, especially those who wished to specialise. The unavailability of opportunities for health personnel to further their training is surprising given that the HRH 2030 strategy expressly identifies the provision of professional training as a key objective^6^. If opportunities do indeed exist, this needs to be communicated to personnel. Previous studies have noted that the rural health professionals encounter difficulties in accessing career development opportunities because of the absence of arranged staff relief, long distances and high costs of travel which prevented access and necessary support for further training^27^.

This study is limited in that it represents the views and experiences of health personnel in one sub-district of KwaZulu-Natal, South Africa. It should be further noted that the interviews were conducted in 2013, early on in the implementation of the South African strategy 2012/13–2015/16. Further research should evaluate the extent to which the objectives of the strategy have been realised. Further, data interpretation was limited through the use of one researcher undertaking the analysis of the data and this may have introduced bias.

## Recommendations

### Living and working conditions

The WHO acknowledges that improving both the living and working conditions of health workers in rural settings can prove costly^2^. However, government must embark on policy initiatives aimed at improving living conditions in the rural areas. This can be done by investing in rural developmental projects that provide safe and decent housing, and other basic infrastructure. There is furthermore an urgent need to formulate strategies to improve the working conditions in the rural areas. Improving the working conditions could be done by ensuring sufficient supply of the basic medical equipment and by ensuring infectious disease control measures are in place.

### Rural allowance

There is a need to revisit the administration and implementation of the rural allowance in order for HRH to feel they are fairly incentivised. This can be achieved by a proper re-definition of the term ‘rural’, to include semi-rural, rural and deep rural and then allocating appropriate allowances to the respective categories. The fact that certain health personnel cadres are excluded from the rural allowance has resulted in resentment. Incentivising all cadres may not be financially feasible but should be investigated.

### Integration of foreign health personnel and human resources for health production

The recruitment of foreign medical doctors has proven successful in alleviating the shortage of medical doctors in rural areas and this practice should continue^28^. However, to ensure proper and effective integration, foreign medical doctors require orientation and support from senior staff and supervisors^28^.

The active recruitment of foreign doctors is potentially a stop-gap measure, and government should ensure an increased production of local HRH. There should be more efforts to ensure that ‘appropriate for rural’ doctors and nurses are produced. This can be done by placing new medical training facilities in provinces with large rural populations while ensuring that the students admitted into existing medical schools are of rural origin, resulting in the likely return to rural practice after graduation^11^. These recommendations build on research undertaken on rural health personnel in other parts of Africa which advocates for the reorientation of training curricula in medical training institutions to address the unique challenges presented by the rural context while also ensuring that health personnel continue to receive the professional support desired^29^.

### Career development opportunities

There is need for medical schools to embrace the use of distance learning and target health professionals working in rural areas. There should also be some career development interaction between specialists and rural health personnel to ensure that they can pursue development opportunities while working in a rural setting. The recently implemented District Support Specialist Teams could be an effective way of fostering this interaction; however, there is a need for continuous review of this initiative to ensure that it achieves this objective.

## Conclusion

The study details the current challenges facing health personnel working in rural areas and the efficacy of selected government strategies aimed at addressing them. Certain government initiatives have been well received by health personnel working in rural areas in KwaZulu-Natal. Specifically, financial incentives have benefited selected health cadres. Further, the augmentation of health personnel with foreign medical and community service doctors has also alleviated the work load burden. Poor working conditions, lack of supervision and inadequate career progression opportunities remain challenges that require attention.

The development and implementation of further government strategies is therefore key to ensuring a consistent supply of health personnel to underserved areas and to ensure improved retention rates. Continuous review of government initiatives is important in ensuring the realisation of their intended outcomes. Lessons can be learnt from the challenges and subsequent responses in other countries. It is therefore vital that the development of future strategies aimed at addressing challenges facing rural health personnel is evidence led.

## Footnotes

1 The purpose of the District Clinical Specialist Teams is to improve both the quality of health care and health outcomes for mothers, newborns and children – that is, the core component of strengthening the district health system (www.health.gov.za).
